# Biocontrol Potential of PeBL2, a Novel Entomopathogenic Bacterium from *Brevibacillus laterosporus* A60, Induces Systemic Resistance against Rice Leaf Folder *Cnaphalocrocis exigua* (Butler) in Rice (*Oryza sativa* L.)

**DOI:** 10.3390/plants11233350

**Published:** 2022-12-02

**Authors:** Khadija Javed, Talha Humayun, Ayesha Humayun, Shahida Shaheen, Yong Wang, Humayun Javed

**Affiliations:** 1 Department of Plant Pathology, Agriculture College, Guizhou University, Guiyang 550025, China; 2 Faculty of Mountain Agriculture and Environmental Sciences, Kohsar University Murree, Murree 47150, Pakistan; 3 Julius Kühn-Institut (JKI) for Biological Control, 64287 Darmstadt, Germany; 4 Department of Surgery, Federal Government Polyclinic Hospital (P.G.M.I), Islamabad 04403, Pakistan; 5 Department of Surgery (Surgical Unit 1 HFH), Rawalpindi Medical University, Rawalpindi 46000, Pakistan; 6 Department of Clinical Studies, Pir Mehr Ali Shah-Arid Agriculture University, Rawalpindi 46300, Pakistan; 7 Department of Environmental Sciences, COMSATS University, Abbottabad 22060, Pakistan; 8 Rothamsted Research West Common Harpenden, Hertfordshire AL5 2JQ, UK

**Keywords:** plant resistance, protein elicitor, PeBL2, *Cnaphalocrocis exigua*, signaling pathway, JA, SA, ET, fecundity, nymphal instars, defense-related gene expressions

## Abstract

The dangerous insect pest known as rice leaf folder *Cnaphalocrocis exigua* (Butler), which reduces rice output globally, twists and feeds on the young rice plant’s leaves. Protein elicitors are hypothesized to be biological components that promote rice in becoming herbivore resistant. The evolving elicitor protein PeBL2, obtained from *Brevibacillus laterosporus* A60, was tested for biocontrol against *C. exigua*. Four distinct PeBL2 doses (74.23, 45.53, 22.26, and 11.13 μg mL^−1^) were assigned to evaluate the impact of PeBL2 on immature growth, survivability, and lifespan. Adult reproductive efficiency and the interaction between the pest and the disease were assessed against *C. exigua*. Further, the assessment of active compounds in PeBL2 with multi-acting entomopathogenic effects investigated the direct correlations of PeBL2 with temperature and climatic change in plants of rice (*Oryza sativa* L.). When compared to controls, PeBL2 treatments reduced the growing population of second- and third-generation *C. exigua*. *Cnaphalocrocis exigua* colonized control plants faster than PeBL2-treated *O. sativa* plants in a host selection test. PeBL2 doses delayed the development of the larval stage of *C. exigua*. PeBL2-treated seedlings generated less offspring than control seedlings, identical to fecundity. Trichomes and wax formation on PeBL2-treated leaves generated an adverse environment for *C. exigua*. PeBL2 altered the surface topography of the leaves, preventing colonization and reducing *C. exigua* reproduction. PeBL2-treated *O. sativa* seedlings exhibited somewhat increased amounts of jasmonic acid (JA), salicylic acid (SA), and ethylene (ET). Systemic defensive processes also included the activation of pathways (JA, SA, and ET). Following these results versus *C. exigua*, the use of PeBL2 in an agroecosystem with integrated pest management and biocontrol appears to be reasonable. These findings shed new light on a cutting-edge biocontrol technique based on *B. laterosporus* A60.

## 1. Introduction

Pathogens must be capable of recognizing and eliminating host defense mechanisms to succeed. Plants have produced resistance (R) proteins in reaction to pathogens that escape, withstand, or reduce basal defense, resulting in gene-for-gene resistance. Plants include a mix of inducible and constitutive defense systems that aid in disease resistance [1]. Microorganisms and pathogens are named using preserved, essential compounds. They are recognized by pattern recognition receptors (PRR) in innate immunity [2]. By increasing extracellular pH, they cause oxidative outbursts and the production of nitric oxide (NO), secondary metabolites, and hypersensitive responses (HR) [1,2]. These defensive mechanisms are initiated by tissue that surrounds the infected site. Consequently, the plant develops systemic acquired resistance (SAR) against several illnesses [3]. Plants are equipped with two defense systems. Flagellin, a pathogen-deterrent molecular pattern (MAMP), for instance, aids plants in detecting microbes and diseases. Molecular patterns associated with microbes or pathogens (PAMP) stimulate the plant’s natural defense mechanism by attaching to a variety of pattern recognition receptors (PRRs) on the plant’s surface [4]. Gene-for-gene resistance is a type of defense that occurs largely inside plants. In this situation, the pathogen-secreted elicitors are analogous to R proteins [5]. As part of effector-triggered immunity (ETI), R proteins induce hypersensitivity responses, oxidative stress, NO production, extracellular pH elevation, cell wall augmentation, and pathogen-related protein expression [3,5]. This sort of reaction starts at the infected area and extends to surrounding cells that are not infected, increasing the plant’s capacity to battle diseases [6].

Elicitors are in charge of boosting the plant’s innate immunity and action strategy in both abiotic and biotic approaches [7]. They help bacteria, viruses, oomycetes, and fungi thrive. A few of these compounds, such as lipids, triggered proteins, and carbohydrates, help plants withstand illness [8,9]. HR and reactive oxygen species (ROS), such as H_2_O_2_ and O_2_, frequently interact via ion influx. These chemicals act as signaling elicitors [10]. They are planting immune units that impact both recipient and non-host plants and can be specific to a plant group or universal [11]. To guarantee food, certain synthetic pesticides may be supplanted by elicitors [12,13,14,15,16,17,18]. Aphid defensive strategies in a variety of aphid–plant associations have been explored. Aphid protection in *Brassica napus* L. (Brassicaceae) reduced immature plant persistence and *Plutella xylostella* (L.) (Lepidoptera: Plutellidae) population increase [19]. Plants respond defensively to ethylene (ET), jasmonic acid (JA), and salicylic acid (SA) [20,21,22]. Numerous studies have revealed that JA and SA have a part in the aphid reaction by boosting gene expression such as *LOX1* (*lipoxygenase*) and *PAL* (*phenylalanine ammonia-lyase*) once aphids feed on them [23].

By promoting host immunity, *B. laterosporus* can reduce infection in plants. Formerly, a protein elicitor named PeBL2 from the *B. laterosporus* strain A60 was reported. The 471 bp PeBL2 gene produces a 17.22 kDa protein with 156 amino acids containing an 84-residue signal peptide [24,25]. However, there are no concrete indications that strain A60 activates protective immunity. In this study, a protein elicitor called PeBL2 from *B. laterosporus* A60 has been identified that could help rice plants develop systemic resistance to *C. exigua*. Systemic resistances and an early plant protection reaction can both be activated by PeBL2. Regional and systemic defensive responses were seen in the host plants following the administration of recombinant PeBL2 from *B. laterosporus* A60 [13,14,26,27]. Plants become more tolerant of it owing to the JA and SA routes. The effect of PeBL2, an elicitor protein made by *B. laterosporus* A60, on the management of *C. exigua* was investigated in the current research. The findings can aid in understanding the systematic resistance mechanism of *B. laterosporus* A60. The current investigation examined the function, mechanism, and biological activity of PeBL2 and its effects on *C. exigua* management. The contents of JA and SA, as well as ET gene expression, were determined following the discovery of trichomes on the leaf surface structure. The purpose of this article is to provide information about PeBL2′s function, method of action, and its impacts on the management of the rice leaf folder (*C. exigua*).

## 2. Results

### 2.1. Expression, Purification, and Evaluation of PeBL2 Elicitor Protein

**Figure 1:**
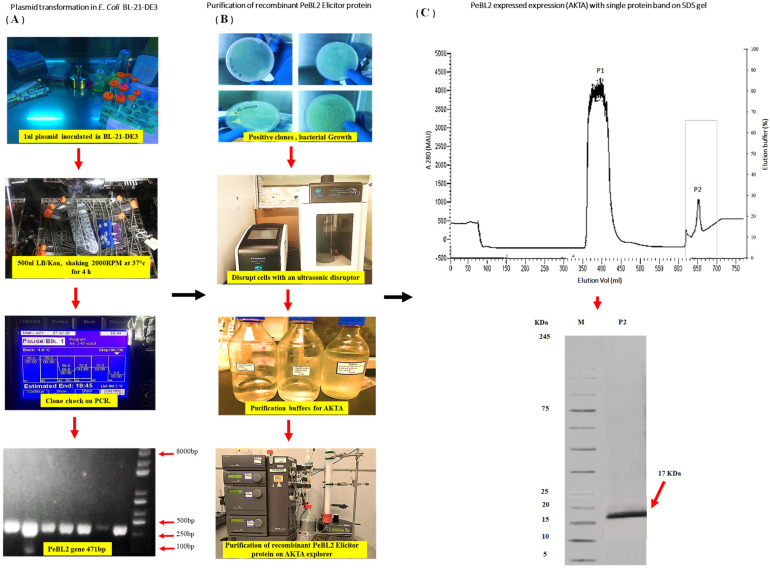
PeBL2, transformation and recombinant protein purification. (**A**,**B**) Evaluation of PeBL2 protein by using the Äkta explorer, His-Trap HP column used for the purification of total *E. coli* proteins. An elution buffer—25 mM Tris, 200 mM NaCl, 500 mM imidazole, and pH 8.0—was used to elute peak P2, which comprised recombinant PeBL2 with a flow rate of 5 mL/min. A HiTrap desalting column was used to load P2. (**C**) The purified and desalted PeBL2 protein formed a single band of the molecular mass of 17 kDa on Tricine SDS-PAGE. Lane M: protein molecular mass marker; lane P2: peak P2.

The pET28 (a+)-TEV/LIC recombinant vector was transformed into *E. coli* BL21 (DE3) cells (Figure 1A). The expressed His6-PeBL2 was soluble in *E. coli* after successful transformation. The full-length DNA sequence encoding PeBL2 (GenBank accession no. ERM16658.1) was amplified from the *B. laterosporus* A60 strain. The length of the PeBL2 gene is 471 bp, encoding a protein with 156 amino acids comprising a theoretical molecular weight of 17.22 kDa. It was confirmed that the PeBL2 is a hypothetical protein. The recombinant protein PeBL2 was purified with the column of the His-Trap HP (GE Healthcare, Waukesha, WI, USA) (Figure 1B) and was desalted, as previously described by Wang et al. [24], in the HiTrap column. A single band showed the characteristics of pure recombinant protein at 17 kDa on Tricine SDS-PAGE (Figure 1C).

### 2.2. Cnaphalocrocis exigua Indoors

**Figure 2:**
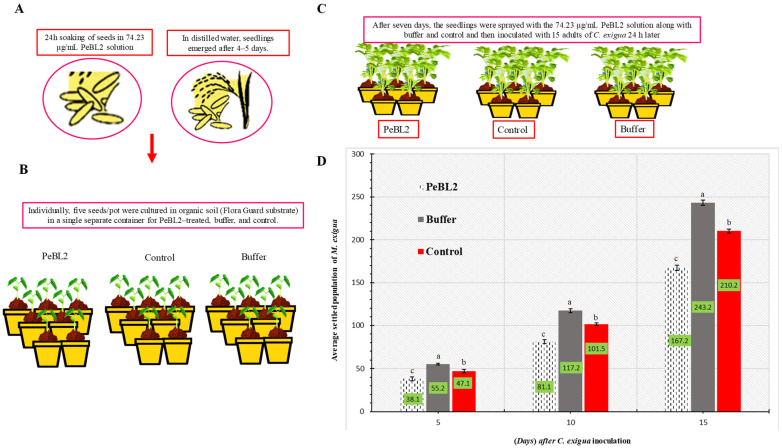
Plants treated with PeBL2, control, and buffer showed *C. exigua* population variations. (**A**,**B**) Seeds and seedling treatment. (**C**) After 7 d seedlings were inoculated with 15 adults of *C. exigua*. (**D**) Seedlings treated with PeBL2 saw a substantial *C. exigua* population loss (mean ± SD). The study used a CRD randomized statistical design and LSD and one-way ANOVA were used to compare data (*p* = 0.05). (a–c) small letters on each bar are significant differences among treatments and control.

**Figure 3:**
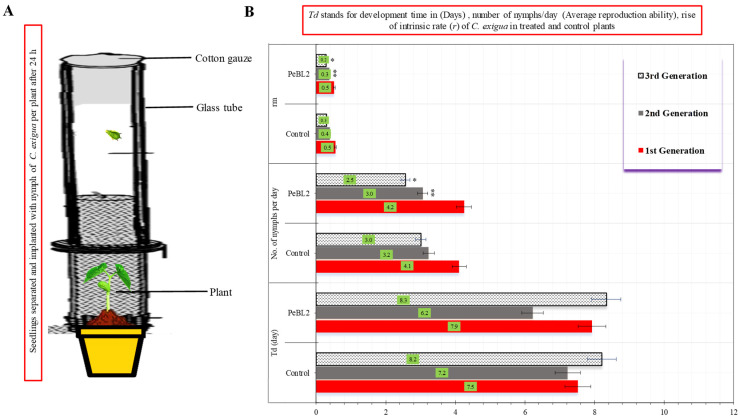
Development time, reproductive capacity, and rate of growth of *C. exigua* (**A**,**B**) in seedlings treated with PeBL2 and control (mean ± SD). The study used a randomized (CRD) statistical design, and SPSS 18.0 was used to compare data by LSD and one-way ANOVA (*p* = 0.05). An asterisk on the bar shows a significant difference from the buffer control as found by students *t*-test (* *p* < 0.05 and ** *p* < 0.05).

Resistance to *C. exigua* was induced by PeBL2 in two separate ways. Predominantly, PeBL2-treated *O. sativa* seedlings exhibited a substantial *C. exigua* population loss; Figure 2 shows the percentage declines in population in the PeBL2 treatment compared with control. *Cnaphalocrocis exigua*’s daily reproducing ability was lowered when fed PeBL2-treated seedlings; all genera showed slower growth, as indicated by the observations that both the second and third generations showed lower growth rates (Figure 3).

### 2.3. PeBL2 Influence on C. exigua

**Figure 4:**
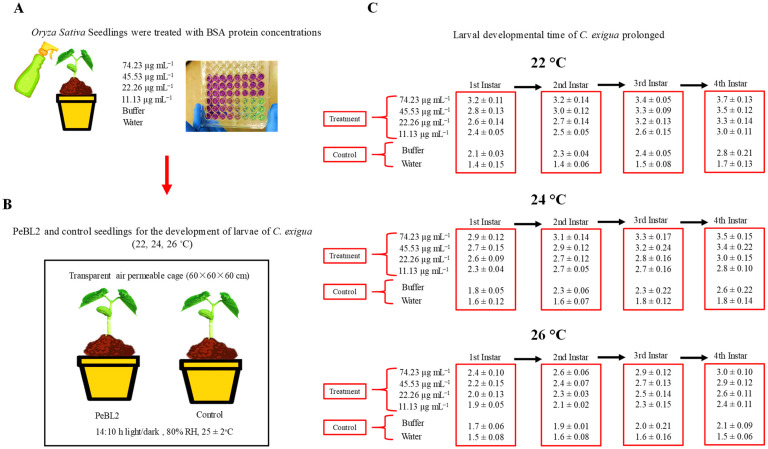
Prolonged larval development time of *C. exigua* instars (1st, 2nd, 3rd, and 4th). (**A**,**B**) *Oryza sativa* seedlings treated with control, buffer, and four PeBL2 protein concentrations prepared by BSA. (**C**) Larval developmental time of *C. exigua* from the prolonged 1st to 4th instar’s in PeBL2-treated *O. sativa* seedlings. Data are shown as the (mean ± SE) of different larval instars of (*C. exigua*) on *O. sativa* plants by the PeBL2 elicitor protein at different concentrations and different temperature regimes (*n* = 10). Data were compared statistically by a factorial ANOVA and LSD at α = 0.05 in Statistix, version 8.1.

*Cnaphalocrocis exigua*’s overall growth duration was impacted by the interactions of varying PeBL2 levels with three temperature regimes. The larval instar development period rose as PeBL2 concentrations grew (Figure 4).

**Figure 5:**
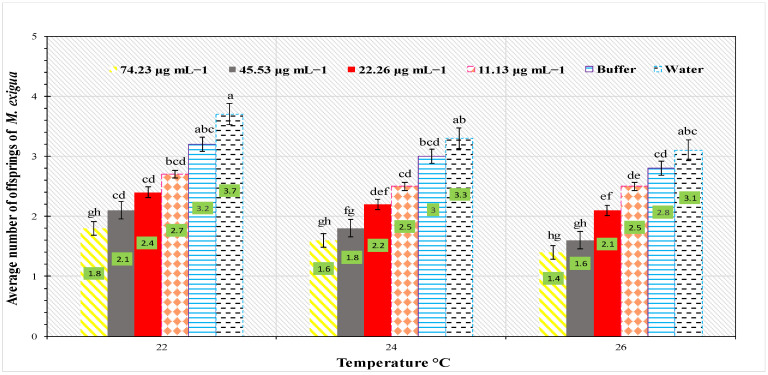
Average fecundity (mean ± SE) of *C. exigua* on *O. sativa* seedlings in relation to various PeBL2 elicitor concentrations, at different temperature regimes (*n* = 10). (a–h) small letters on each bar are significant differences among treatments and control, Data were compared statistically; the LSD, ANOVA, and Leven’s test were employed by Statistix version 8.1 at α= 0.05).

Fourth larval instar development time was 3.7 days at 74.23 μg mL^−1^ at 22 °C, and a dosage of 11.13 μg mL^−1^ at 26 °C yielded a minimal larval growth time of 1.9 days. *Cnaphalocrocis exigua* fecundity had a substantial effect on PeBL2 concentrations and temperature regimes (Figure 5). The experiment discovered that fecundity was minimal at a maximum temperature of 26 °C and greatest at a minimum temperature of 22 °C.

### 2.4. PeBL2 Impact on O. sativa

**Figure 6:**
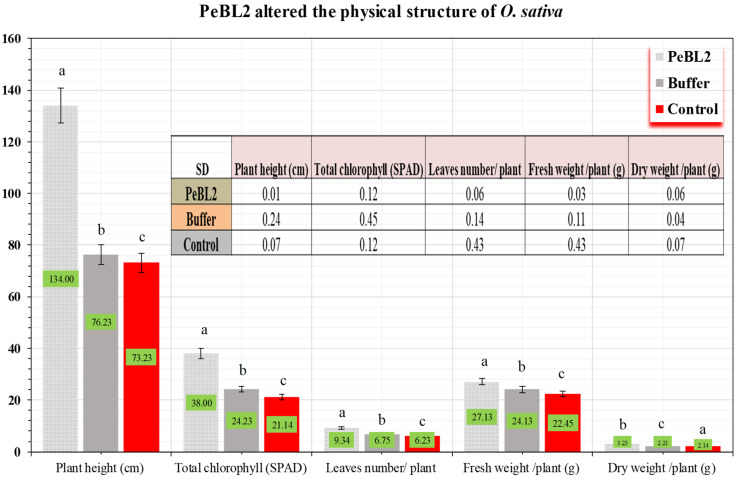
Effect of PeBL2 on growth of PeBL2-treated and control seedlings of *O. sativa*. Data shown in mean (± SD) of PeBL2, buffer, and control seedlings (*n* = 10). (a–c) small letters on each bar are significant differences among treatments and control. The data were compared using LSD, one-way ANOVA, and Levene’s test in SPSS 18.0. *(p* = 0.05).

Compared to control seedlings, PeBL2 substantially impacted the plant growth and surface structure of *O. sativa* seedlings (Figure 6). There were substantially higher trichomes in PeBL2-treated seedlings than in control seedlings, with 79.10 ± 0.21 mm^−2^ in PeBL2-treated vs. 22.14 ± 0.10 mm^−2^ in control seedlings (*p* = 0.05). With a better surface environment and a more complex wax structure, *C. exigua* colonization should be more difficult.

### 2.5. Concentration of SA, JA, and ET

**Figure 7:**
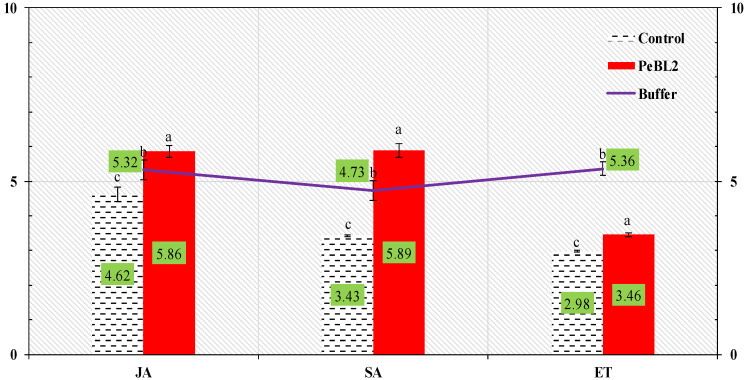
JA, SA, and ET contents in *O. sativa* seedlings (mean ± SD). PeBL2-treated tests were gathered 1 d after the spraying. *Cnaphalocrocis exigua* were inoculated on seedlings 1 d after they were sprayed with treatment, and samples were collected 1 d after they were inoculated: (a–c) are significant differences among treatments and control. Data were compared statistically. The LSD, ANOVA, and Levene’s test were employed by Statistix version 8.1. (*p* = 0.05).

On PeBL2, JA, SA, and ET investigate the relationships between cuticular wax depositing, trichome quantity, and *C. exigua* invasion. Higher levels of JA, SA, and ET were discovered in PeBL2 saplings (Figure 7). All three molecular mechanisms are required to form *C. exigua* resilience in *O. sativa*. In line with the findings, the protein elicitor evoked an intrinsic immune or protective activity in *O. sativa* plants.

### 2.6. Relative Fold Changes in the Expressions of Defense-Related Genes

**Figure 8:**
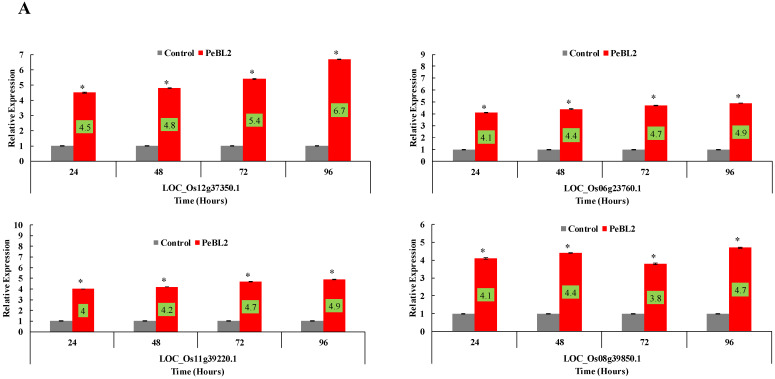

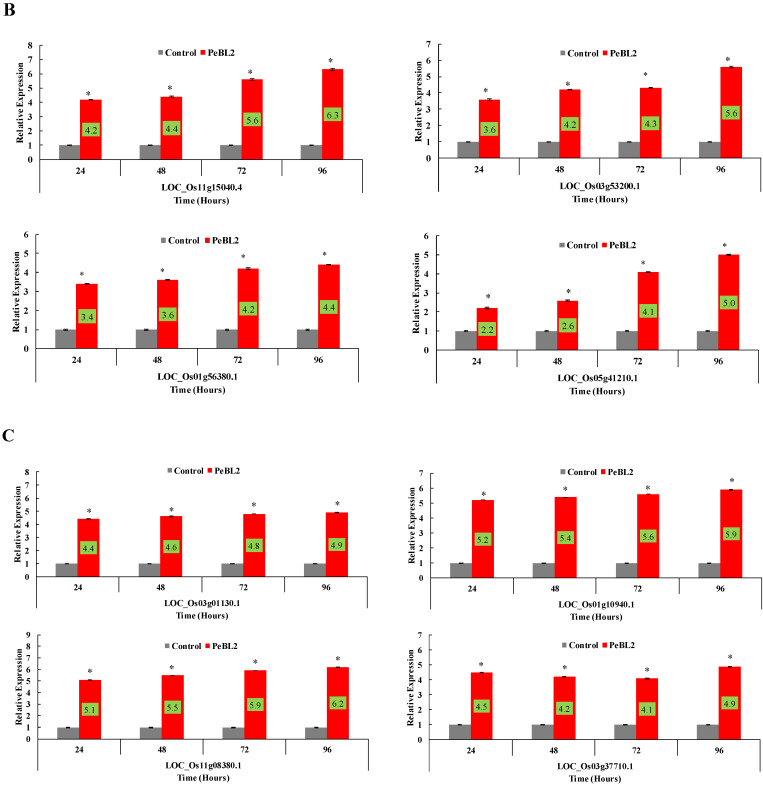
Relative expression of plant defense from JA (**A**), SA (**B**), and ET (**C**) pathways found at various time intervals after treatment with PeBL2 elicitor and aphid infestation. For each gene, an asterisk on bar shows a significant difference from control by Student’s *t*-test (*p* < 0.05).

PeBL2 increased the defense response in *O. sativa* seedlings. All test genes were upregulated by PeBL2 treatment, showing transcripts statistically more expressed than in the control. It was considered that induced resistance was caused by the aphid infestation and enhanced by PeBL2. Although genes involved in the JA pathway were moderately expressed, all JA-associated genes were upregulated after 24, 48, 72, and 96 h of *C. exigua* infestation (Figure 8A). Similar trends were observed for all SA- and ET-associated genes that were significantly upregulated and significantly different from control samples for all observation times (Figure 8B,C). This suggests that resistance against *C. exigua* was due to increased transcription of the JA, SA, and ET genes.

## 3. Discussion

Elicitors are a novel biological tool for pest management that play a function in the signaling system and crop protection [14,28]. Several *B. laterosporus* strains have demonstrated various broad-spectrum anti-microbial activities, acting as anti-microbial peptides in microbes such as bacteria and fungi. They can enter into the cell and transfer to the cytoplasm and nucleus to interrupt protein synthesis by mixing up DNA and RNA [28]. PAMPs and MAMPs are widespread in fungi and pathogenic bacteria that are either necrotrophic or biotrophic [29]. The possible activities of PeBL2 derived from *B. laterosporus* strain A60 were demonstrated in this study for *M. exigua* insect pest management. Certain studies have previously shown chemical elicitors significantly reduced the activity of herbivorous pests in cucumber crops by applying chemical elicitors such as methyl jasmonate, benzothiadiazole, and other plant defenses, including proteinase inhibitors [30]. Results from this study confirmed previous results that the use of a methyl salicylate elicitor reduced the soybean aphid *Aphis glycines* Matsumura by up to 40% [29,30]. Here, bioassays showed that population development on PeBL2-treated *O. sativa* plants was significantly slower compared to the buffer and control. Previous studies have shown a negative influence of exogenous applications of elicitors, including methyl jasmonate (MJ) and benzothiadiazole (BTH), on the population growth and fitness of different aphid species, an effect confirmed by the present findings [30]. Similarly, a biocontrol potential was discovered against various Diptera, Coleoptera, and Lepidoptera, as well as against nematodes and mollusks [31]. The current study presented the capability of PeBL2 to overwhelm herbivores by swaying population and growth parameters. Trichomes are the first line of physical resistance to pathogenic microorganisms and herbivores. These hairy adjuncts of plant epidermal cells affect the herbivores’ behavior, and the density role of trichomes in *Solanum* spp., i.e., seven trichomes with two major defense-related effects, have been tested [31]. First, a plant surface represents a physical barrier because its thick matte hair provides energy, limits feeding capacity, and reduces access to the surface by insects. Excessively hairy plants, such as *Solanum hirsutum*, are avoided by *C. exigua*. Trichomes are also associated with the basic defensive mechanism of the tomato plant, as the surface area covered by the epidermal cell appendages of unicellular or multicellular hairs provide resistance to a variety of pests due to the “pubescence” of a plant. Colorado potato beetle (*Leptinotarsa decemlineata*) (Coleoptera: Chrysomelidae) settlement with thick trichomes was reduced in soybeans compared with the trichome-removed plants, which attracted more beetles [32]. PeBL2 abridged disease rigorousness, triggering a photosynthesis process that mirrored the characteristics of growth of plants [33], and it enhanced induced resistance in the PeBL2-treated *O. sativa* seedlings as well. Compared to the controls, the PeBL2-treated seedlings and leaves had more trichomes. The PeBL2-treated *O. sativa* seedlings and leaflets were reported to have inhibited the reproduction and settlement of *C. exigua* with an increased number of trichomes. Another key part of the physical barrier lignin is the cell wall, which underpins plant resistance and is an indicator for the improvement of the cell wall [33]. The physical defenses of plants include, in response to biotic and abiotic stress, trichomes and wax production. Their establishment can be induced by direct damage, e.g., as induced by leaf-cuts, methoxyfenozide, and manganese [34]. The use of exogenous phytohormones, methyl jasmonate (MJ), or JA can also affect cuticular wax deposition and trichome density, as shown in *Arabidopsis* and tomatoes, respectively [35]. The SA application was due to wax deposition in *Brassica napus* [36]. Accumulations of SA, JA, and ET in PeBL2-treated *O. sativa* plants can, therefore, be hypothesized as being related to increased trichome density and the deposition of cuticular wax. In addition, the treatment of the PeBL2 elicitor had adverse effects on *C. exigua* fecundity. PeBL2-treated *O. sativa* plants produced significantly fewer *C. exigua* than the buffered and controlled seedlings. The results were consistent with previous studies showing that exogenous SA and MJ have caused lower mean lifetime fecundity [29,35,36]. Therefore, optimum temperatures (e.g., 22 °C) demonstrated a maximum *C. exigua* fecundity, with the minimum fecundity at higher temperatures (26 °C) due to a decreased metabolic rate [37]. Similarly, a variance analysis showed that in PeBL2-treated *O. sativa* plants, the development time of larvae was extended compared to the control; even at a lower temperature (22 °C), the maximum larval development time was observed, indicating that a one-degree temperature increase affected the life cycle of the insect [38]. Additional studies need to be conducted to understand the underlying mechanism of PeBL2 in *O. sativa*, in particular its effect on fecundity and larval development time.

Additionally, JA, SA, and ET increased marker gene transcriptions, signaling that they play an essential role in *M. exigua* resistance. After *C. exigua* infestation in *Arabidopsis*, the transcript genes *LOC_Os12g37350.1, LOC_Os11g39220.1, LOC_Os06g23760.1, LOC_Os08g39850.1, LOC_Os11g15040.4, LOC_Os01g56380.1, LOC_Os03g53200.1, LOC_Os05g41210.1, LOC_Os11g08380.1, LOC_Os03g01130.1, LOC_Os01g10940.1,* and *LOC_Os03g37710.1* were significantly increased. JA, SA, and ET molecules impart resistance to insect herbivorous diseases and pathogens, which enhances plant defense responses [39]. All JA, SA, and ET test genes showed significant and robust regulation. *Oryza sativa* grew increasingly resilient to *C. exigua* after being introduced to PeBL2. Beneficial microorganisms initiated systemic resistance. This reaction is governed by a signaling system that involves the plant hormones SA, JA, and ET, as well as by microorganisms [40]. According to some evidence, the SA, JA, and ET pathways collaborate to alter how well the plant reacts to various diseases [40,41]. These plant coping strategies defend against key agricultural pests [41]. The scientists discovered that PeBL2 boosted the levels of JA, SA, and ET-responsive genes via relationships with TGA transcriptional regulators to activate PR genes [42]. The results suggest that relative protein expression influences PeBL2-mediated system-wide protective reactions in *O. sativa*. Secondary metabolite formation can assist plants in resisting infestation by building mechanical obstacles to microbial activity, which drives phenolic metabolism and lignin creation, according to a previous study by Chaerle et al. [43]. Furthermore, phenolic substances such as scopoletin and phenolic acids can reinforce the cell wall, rendering it much more resistant to fungi and bacteria. When phenyl propanoid esters are cross-linked with ferulic acid, auto luminescent lignin-like polymers such as hydroxycinnamic acids and their derivatives are formed [44]. Systemic responses are engaged when an aphid infests plants [45]. These findings indicate that our research can aid us in better comprehending how PeBL2 from *B. laterosporus* A60 acts in *O. sativa* to control *C. exigua* [46].

## 4. Materials and Methods

### 4.1. Colonies of Plant, Insect, and Bacterial Culture

This study aimed to raise populations of *C. exigua* and *O. sativa* in a regulated growing period before the testing. *Cnaphalocrocis exigua* were collected from a *Triticum aestivum* L. field in Guiyang, Guizhou, China, and shifted to *O. sativa* seedlings. A colony of *C. exigua* was maintained at ambient temperature for six months before the experiment. *Cnaphalocrocis exigua* were raised on rice plants at a temperature of 25 ± 2 °C, 80% humidity levels, and a photoperiod of 10 D:14 L. *Oryza sativa* seeds were rinsed in 75% ethanol for 20–40 s. Before use, the item was water cleansed and submerged for two to three days. Isolate of *B. laterosporus* strain A60 was maintained on solid media of potato dextrose agar (PDA: 20 g mL^−1^ agar, 20 g mL^−1^ dextrose, and 200 g mL^−1^ potato) and LBA agar (5 g mL^−1^ yeast extract, 10 g mL^−1^ tryptone and NaCl each, and 15 g mL^−1^ agar). The Luria–Bertani (LB) medium was utilized for bacterial *B. laterosporus* strain A60, which was grown in 200 mL LB medium for 48 h at 30 °C and 180 rpm for protein isolation.

### 4.2. Isolation and Detection of Crude Protein

Culture broth was centrifuged, and the supernatant was added to ammonium sulfate to 62% relative saturation (*w*/*v*). The sample was centrifuged at 12,000× *g* for 20 min at 4 °C and the pellet was collected and dissolved in 100 mL of buffer (25 mM MES-NaOH, pH 6.2). It was further dialyzed using the same buffer for 48 h for removing ammonium sulphate and harvesting the precipitate. Insoluble debris was removed by centrifuging and crude protein was finally collected by freeze-drying.

### 4.3. Protein Purification

ÄKTA Explorer 10 protein purification system (GE Healthcare, Piscataway, NJ, USA) (Amersham Biosciences) was used to purify the crude protein. The crude protein was loaded onto a HisTrap SPHF column (5 mL; GE-Health, Waukesha, WI, USA). Briefly, washing of column was conducted with Buffer A for eluting bound proteins and removing traces of any unbound proteins. An increasing linear gradient NaCl (0–1 M) was used to elute the target proteins. Buffer A was used to monitor the elicitor activity against the *O. sativa* plant leaf in each fraction after dialysis [24,25]. Furthermore, HPLC was conducted for purification of the crude proteins. Positive fractions were separated using a solution of n-butyl alcohol, isopropyl alcohol, water, and aqueous ammonia (60:20:20:10). The concentration of ACN (acetonitrile) was raised in the eluted solvent from 10 to 60% (v/v) for 28 min by using increasing linear gradient at a flow rate of 0.2 mL/min. T3 C18 (Waters Atlantis) reverse phase column was equilibrated (2.1 × 150 mm, 3.5 m, 40°C) with 2 mM NH4FA/0.1% FA/water and 5% ACN and the pooled active fraction was injected onto the column. Through the fraction collector, the sample was collected, coupled with the monitoring of absorbance which was carried at 235 nm. To determine the purity, each peak was tested against *O. sativa* plant leaves, while the fraction exhibiting elicitor activity was chromatographed.

### 4.4. Mass Spectrometry Analysis

Using the Triple TOF 5600 TOF MS Analyser (Applied Biosystems SCIEX, Concord, ON, Canada), the active segment collected after preparative HPLC was analyzed. MS scanning mode with a scan range of 250–2000 (*m*/*z*) was used to acquire data. Samples were digested after lyophilization using 2 μL of a 20% nitrile reconstitution. A 1 μL aliquot of dissolved sample was added directly to the illustration point on the target. After allowing the solvent to dry naturally, 0.5 μL of over-saturated CHCA Matrix Solution (solvent for 50% ACNO, 1% TFA) was added to the corresponding target and dried. Nitrogen was blown onto the target sample and then the sample was put into the instrument through the target trough; analysis was completed using a time-of-flight tandem mass spectrometer 5800 MALDI-TOF/TOF, Applied Biosystems SCIEX, Concord, ON, Canada). To test the analysis, the laser source was 355 nm wavelength of Nd: YAG Laser. The acceleration voltage was 2 kV, and positive ion mode and automatic data acquisition were used. The first-order mass spectrometry scan range was 800–4000 Da. A signal-to-noise ratio greater than 50 was selected for the two-stage mass spectrometry of mother ions (MS/MS) analysis. Each illustration click was based on the selection of an 8A mother ion, a two-grade mass spectrometry (MS/MS) cumulative overlay of 2500, time, and a collision energy of 2 kV with CID shut down. Using the NCBI taxonomy database against Brevibacillus laterosporus (40021), a combined (MS + MS/MS) enzyme search was carried out with the following parameters: trypsin-fixed modifications, carbamidomethyl (C); dynamical modifications, oxidation. The mass values were as follows: Monoisotopic Protein Mass, unrestricted peptide; Mass Tolerance, ± 100 ppm fragment; Mass Tolerance, ± 0.4 Da peptide; Charge State, 1+ ax; Missed Cleavages, 1.

### 4.5. Gene Cloning

From the results of de novo sequencing and BLAST searches in the NCBI (www.ncbi.nlm.nih.giv/perotein/ERM166658.1, accessed on 18 August 2022) database, a peptide sequence was obtained. The primers (F primer, 5′-TCAACCACATCCTCCGTACAGC-3′ and R primer, 5′-TGCTACGAACCAGAGAAGCCA-3′) were designed to amplify the entire coding sequence of elicitor protein-encoding gene of *B. laterosporus*. The genomic DNA was isolated using the Bacterial DNA Kit D3350-01 (ΩMEGA Biotech). The elicitor-encoding gene was amplified using genomic DNA as a template. The purified fragment was cloned into a pMD18-T simple vector (Takara Biotechnology, Dalian, China) and then transformed into Trans 1 T1 (Transgene Biotech, Beijing, China) for sequencing. The gene was constructed into the expression vector, pET-28-a(+), using the BamHI and XhoI restriction sites. The recombinant vector was then transformed into *E. coli* BL21 (DE3) for protein expression. The recombinant novel protein was induced by the addition of 0.5 mM isopropyl β-D-1-thiogalactopyranoside (IPTG) (Sigma, St. Louis, MO, USA). The cells were collected by centrifugation and then disrupted by an ultrasonic disruptor to obtain the supernatant. The purification of the recombinant protein consisted of three sequential procedures: (1) affinity chromatography with a HisTrap HP affinity column (GE Healthcare, Piscataway, NJ, USA), (2) ion exchange chromatography with a Resource Q column (GE Healthcare, Piscataway, NJ, USA), and (3) size exclusion chromatography with a Superdex-200 column (GE Healthcare, Piscataway, NJ, USA). By separating on SDS-PAGE and staining the polyacrylamide gel with Coomassie Brilliant Blue, the protein purity and molecular weight were determined. Protein molecular markers (Ferments, Hanover, NH, USA) were used to estimate the size of the purified protein.

### 4.6. Expression and Purification of the Recombinant PeBL2 Elicitor Protein

PeBL2 was produced with the recombinant vector pET-28-aTEV/LIC in *Escherichia coli* BL21-DE3 (Novagen, Darmstadt, Germany). The pellets were removed, and the supernatant cells were resuspended and sonified by the ultrasonic disruptor. The supernatant was collected and filtered with filter paper (size 0.22 µm) after the solution was centrifuged at 12,000 rpm for 15 min. The Äkta Explorer Protein Purification System (Amersham Biosciences, Temecula, CA, USA), as described by Wang et al., 2015, Jatoi et al., 2019, Javed et al., 2020, and Javed and Qiu, 2020 [13,14,24,25], with a His-Trap HP column (GE Healthcare, Waukesha, WI, USA), used various loading buffers (A, B, C, and D) for the further purification of the elicitor protein PeBL2. Buffer A (50 Mm Tris-HCl, 8.0 pH) washed off other elicitors from the column quickly, and Buffer B was used to stabilize the column (50 Mm Tris-HCl, 200 Mm NaCl). For the solution elution elicitor protein, Buffer C (50 Mm Tris-HCl, 200 Mm NaCl, and 20 Mm imidazole, pH 8.0) and elusion Buffer D (50 Mm Tris-HCl, 200 Mm NaCl, and 500 Mm imidazole, pH 8.0) were used. Then, the PeBL2 elicitor protein was desalted in a HiTrap desalting column (GE Healthcare, Waukesha, WI, USA), as described by Wang et al., 2015 and Jatoi et al., 2020 [24,25]. The molecular mass of the purified elicitor protein was measured by a 12% SDS-PAGE resolving gel, and a GenStar M223 protein marker (~5–245 kDa) was used for the estimation of the molecular mass of the purified PeBL2 elicitor.

### 4.7. Cnaphalocrocis exigua Infestation on the Plant

This experiment was designed to estimate the size of the *C. exigua* population that had settled. *Oryza sativa* plants were soaked in 74.23 μg mL^−1^ PeBL2 for one day. Four organic seeds were developed (Flora Guard substrate). After seven days, *O. sativa* seedlings were sprayed with 74.23 μg mL^−1^ PeBL2 solution and infested with 15 *C. exigua* female adults 24 h later. Seedlings were treated weekly. Every five days after inoculation, *C. exigua* were counted. The data fractions were used for data analysis and the conclusion of results. Controls and negative controls were treated with water and 74.23 μg mL^−1^ of a buffer (50 mM Tris-HCl, pH 8.0), respectively. Plants were caged (60 × 60 × 60 cm) in transparent, breathable mesh. Each time, four replicates were used.

### 4.8. Cnaphalocrocis exigua Development

This experiment aimed to examine if nourishing PeBL2-treated or control seedlings increased *C. exigua*’s intrinsic development. *Oryza sativa* seedlings were treated the same way (Sec: 2.7). After one day, the seeds were showered with 74.23 μg mL^−1^ of PeBL2 pure protein solution. A glass tube wrapped with cotton gauze segregated sprouting, and *C. exigua* mortality on the leaves was confined in a plastic ecological cage (Cangzhou Hengyun Plastic Industry, Cangzhou, Hebei Province China) (2.7 × 2.7 × 2.7 cm). The ecological cage’s periphery was sponge-coated to eliminate mechanical damage to the leaf. The *C. exigua* instars 1st–4th were tested for larval development for 12 h. To avoid overcrowding, newly molted larvae were tallied twice daily to govern the overall aggregate of period and offspring produced. This was repeated on seeds and plants five days afterwards. The experiment employed 30 replicates of each treatment. The intrinsic rate was determined in the following manner:

*r_m_* = 0.738 × (ln *Md*)/*Td*

*Md* is the number of newborn larvae within a development period of *Td*, which is the time interval between a *C. exigua* birth and reproduction

### 4.9. Cnaphalocrocis exigua Bioassay

This study examined the larval development time as well as the fecundity of *C. exigua*. On *O. sativa* plants, PeBL2 was tested against *C. exigua* at 74.23, 45.53, 22.26, and 11.13 μg mL^−1^. This was calculated using the Bradford assay [25]. Using a separate spray bottle, approximately 3 mL of PeBL2 was sprayed into the *O. sativa* plants at the three-leaf stage. Water and buffer were used to treat the controls (50 mM Tris-HCl, pH 8.0). Then, 3–6 freshly molted larvae of *C. exigua* were given to *O. sativa* plants and desiccated instantaneously. The entire number of descendants formed by all *C. exigua* instars was used to calculate the overall larval development period, while *C. exigua* longevity was derived from the number of days they lived. The bioassays were performed in triplicate at three different temperatures (22, 24, and 26 °C).

### 4.10. PeBL2 Influences on C. exigua

This research aimed to determine if PeBL2 influenced *C. exigua* development and morphology. *Oryza sativa* seedlings and seeds were treated as intended in Section 2.3. For two days, samples were collected using a 3.5 % glutaraldehyde buffer in 0.1 M phosphate solution (pH 7.2). All samples were submerged in 1% osmic acid for five total hours. They were subjected to a 15 min ethanol gradient ranging from 100% to 95% to 90% to 80% to 70% to 30%. Leica’s EM critical point drier dehydrated all crucial points (CPD030; Leica Bio-systems, Wetzlar, Germany). A Hitachi H-7650 TEM was used to assess all of the specimens. In ten copies, PeBL2-treated colonies were recorded.

### 4.11. SA, JA, and ET Quantity with HPLC/MS

The point of the study was to figure out how much SA, JA, and ET were produced in this manner; seeds and seedlings were managed as initially reported. The aerobatic sections of seeds were obtained for SA, JA, and ET [26,27,47]. This was accomplished using an HPLC/MS (Shimazu Research Instruments, ODS-C18, 3 m, and 2.1 per 150 mm Kyoto, Japan). In HPLC, methanol liquid stage, 60% concentration, and a sample temperature of 4 °C were utilized. A negative ion system was simulated using the different indicators: solvents 250 °C, heating blocks 200 °C, air flow rate 10 L/min, nebulizing gas 1.5 L/min, detector potential 1.30 kV, interfaces 3 kV (SA m/z: 137.00; JA: 209.05).

### 4.12. Isolation of RNA and cDNA Synthesis

Total RNA of the treated and control plant leaves after feeding of rice leaf folder was isolated separately using the plant RNA ER301-01 kit (Trans Gen Biotech, Beijing, China), following the manufacturer’s protocol. The concentration of RNA was quantified using a nano-photometer (NP80 Touch, Implen Inc., Westlake Village, CA, USA). A One-Step cDNA Removal and cDNA Synthesis AT341-01 kit (TransGen Biotech, Beijing, China) was used for cDNA synthesis.

### 4.13. Real-Time Quantitative Reverse Transcription PCR (qRT-PCR)

The goal of this experiment was to determine the relative expression levels of genes. To extract RNA, synthesize cDNA, and perform a real-time quantitative polymerase chain reaction (qRT-PCR) (ABI 7500 Real-Time PCR System), kits from TransGen Biotech (Beijing, China) were utilized. qRT-PCR was performed to quantify the expression profile of the key genes associated with JA, SA, and ET pathways. The feeding of rice leaf folder on plants treated with protein elicitors was considered a treatment, and plants treated with buffer were considered controls. For amplification of qRT-PCR, 12 gene primer pairs (Table 1) were used with the Applied Biosystems, USA (ABI 7500) system. All reactions were performed using the SYBR Premix Ex Taq II kit (TransGen Biotech, Beijing, China) in a 20 μL total sample volume (2.0 μL cDNA,10.0 μL SYBR Premix Ex Taq II, 1.8 μL of primers, and 6.2 μL of distilled deionized water). The qRT-PCR procedure was as follows: pre-denaturation at 95 °C for 10 min, denaturation at 95 °C for 15 s, and annealing at 60 °C for 15 s, with a total of 40 cycles. Standard curves were run simultaneously. An NP80 nano-photometer was used to assess the purity and excellence of the RNA. The *18S* was considered as endo-reference [48]. *LOC_Os12g37350.1, LOC_Os11g39220.1, LOC_Os06g23760.1, LOC_Os08g39850.1, LOC_Os11g15040.4, LOC_Os01g56380.1, LOC_Os03g53200.1, LOC_Os05g41210.1, LOC_Os11g08380.1, LOC_Os03g01130.1, LOC_Os01g10940.1,* and *LOC_Os03g37710.1* were chosen as key genes [49]. The key genes involved and their respective primers are listed in Table 1. The 2^-ΔΔCT^ methodology was used to determine the relative expression levels of genes [50].

### 4.14. Data Examination

Data from two treatments were compared using an independent Levene’s test and a two-tailed *t*-test, whereas data from three or more treatments were compared using the LSD and an ANOVA with Statistix software version 8.1 (Tallahassee, FL, USA). Prior to analysis, *C. exigua* fecundity data were square root transformed. A one-way factorial analysis of variance was performed among treatment factors such as the PeBL2 concentrations and varied temperature regimes to eliminate differences, followed by a 95% probability LSD test. The gene expressions (qRT-PCR) were obtained by the comparative CT (2^-ΔΔCT^) method; Student’s *t*-tests (α = 0.05) were used to compare the fold changes in plant samples treated with PeBL2 and buffer.

## 5. Conclusions

PeBL2 enhanced *C. exigua* resistance in *O. sativa*, reducing second- and third-generation *M. exigua* fecundity and its colonization. PeBL2 affected the surface structures of *O. sativa* leaves. SA, JA, and ET exhibited slight increases in relative expression levels, acting as potential factors engaged in systemic defense responses generated by PeBL2 in *O. sativa*. Furthermore, PeBL2 exhibited a significant suppression of *C. exigua* life characteristics in the laboratory; however, more research is required to maximize its effects in the field. Future research should explore whether the wax composition affects *C. exigua* behavior, how SA and JA work in induced resistance, and whether other plant hormones are involved. We are convinced that PeBL2 could be used as a “vaccine” for *O. sativa* seeds and seedlings to protect them from *C. exigua*.

## Figures and Tables

**Table 1:** Primers for all test genes of JA, SA, and ET.

JA/SA/ET Pathways	Test Genes	Forward Sequence (5′…… 3′)	Reverse Sequence (5′…… 3′)
JA	*LOC_Os12g37350.1*	CTCCATGGTTGGTGGAACGA	TAGGGGTACTGGCCGAAGTT
JA	*LOC_Os11g39220.1*	GCTCACACTTGCGGAATCAC	GGCTTTGTTTGGGGCAACAT
JA	*LOC_Os06g23760.1*	AGCTCAGGTCACCGACTTTG	ATGAAACGGGAATTCGGCCT
JA	*LOC_Os08g39850.1*	GAGATGAGGAGTTCGCGAGG	ACGGCAAGAAGAGGTCATGG
SA	*LOC_Os11g15040.4*	TTCAATGCAGGAGGGACGAC	AGTCATGCATGCGGTTCTCA
SA	*LOC_Os01g56380.1*	GCATCAACGTCGTGCCTTTC	GATCGGAGCAGTAGACGACG
SA	*LOC_Os03g53200.1*	TCTTCGACAAGAACGGCGAT	AGGCCAAGAGAACGAGTCAC
SA	*LOC_Os05g41210.1*	GCGACGGTTGCATCACTACT	GCCTCAGTTGGGTTCTGACC
ET	*LOC_Os11g08380.1*	TAGCAATGGCCGCTTCAAGA	CTTGAAGCTCGGGTAGTCGG
ET	*LOC_Os03g01130.1*	GCGGAGCTGTACCTCAACAT	CTTGGAAGACTCCGCTGGTT
ET	*LOC_Os01g10940.1*	CGGAGACGTTCCTCTTCACC	CTTCTCGTAGTCGACGCTGG
ET	*LOC_Os03g37710.1*	TGAGAGGAGCCATAGGTGGT	GTAGCGGCTCATGTCGAAGT
	*18S*	GTGACGGGTGACGGAGAATT	GACACTAATGCGCCCGGTAT

## References

[1] Silipo A., Erbs G., Shinya T., Maxwell Dow J.M., Parrilli M., Lanzetta R., Shibuya N., Newman M.A., Molinaro A. (2009). Glycoconjugates as elicitors or suppressors of plant innate immunity. Glycobiology.

[2] Ma W., Berkowitz G.A. (2007). The grateful dead: Calcium and cell death in plant innate immunity. Cell. Microbiol..

[3] Garcia-Brugger A., Lamotte O., Vandelle E., Bourque S., Lecourieux D., Poinssot B., Wendehenne D., Pugin A. (2006). Early signaling events induced by elicitors of plant defenses. Mol. Plant-Microbe Interact..

[4] Nürnberger T., Brunner F. (2002). Innate immunity in plants and animals: Emerging parallels between the recognition of general elicitors and pathogen-associated molecular patterns. Curr. Opin. Plant Biol..

[5] Thomma B.P.H.J., Nu T., Joosten M.H.A.J. (2011). Of PAMPs and Effectors: The Blurred PTI-ETI Dichotomy. Plant Cell.

[6] Yano A., Suzuki K., Uchimiya H., Shinshi H. (1998). Induction of hypersensitive cell death by a fungal protein in cultures of tobacco cells. Mol. Plant-Microbe Interact..

[7] Zhao L.Y., Chen J.L., Cheng D.F., Sun J.R., Liu Y., Tian Z. (2009). Biochemical and molecular characterizations of *Sitobion avenae*-induced wheat defense responses. Crop Prot..

[8] Ellis J.G., Rafiqi M., Gan P., Chakrabarti A., Dodds P.N. (2009). Recent progress in discovery and functional analysis of effector proteins of fungal and oomycete plant pathogens. Curr. Opin. Plant Biol..

[9] Bent A.F., Mackey D. (2008). Elicitors, effectors, and R genes: The new paradigm and a lifetime supply of questions. Annu. Rev. Phytopathol..

[10] Foyer C.H., Noctor G. (2005). Oxidant and antioxidant signalling in plants: A re-evaluation of the concept of oxidative stress in a physiological context. Plant Cell Environ..

[11] Montesano M., Brader G., Palva E.T. (2003). Pathogen derived elicitors: Searching for receptors in plants. Mol. Plant Pathol..

[12] Hael-Conrad V., Perato S.M., Arias M.E., Martínez-Zamora M.G., Di Peto P.D.L.Á., Martos G.G., Castagnaro A.P., Díaz-Ricci J.C., Chalfoun N.R. (2018). The elicitor protein AsES induces a systemic acquired resistance response accompanied by systemic microbursts and micro-hypersensitive responses in *Fragaria ananassa*. Mol. Plant-Microbe Interact..

[13] Javed K., Javed H., Qiu D. (2020). Biocontrol Potential of Purified Elicitor Protein PeBL1 Extracted from *Brevibacillus laterosporus* Strain A60 and Its Capacity in the Induction of Defense Process against Cucumber Aphid (*Myzus persicae* ) in Cucumber (*Cucumis sativus*). Biology.

[14] Javed K., Qiu D. (2020). Protein Elicitor PeBL1 of *Brevibacillus laterosporus* Enhances Resistance Against *Myzus persicae* in Tomato. Pathogens.

[15] Javed K., Talha H., Ayesha H., Wang Y., Javed H. (2021). PeaT1 and PeBC1 Microbial Protein Elicitors Enhanced Resistance against *Myzus persicae* Sulzer in Chili *Capsicum annum* L.. Microorganisms.

[16] Javed K., Javed H., Qiu D. (2021). PeBL1 of *Brevibacillus laterosporus* a new biocontrol tool for wheat aphid management (*Sitobion avenae*) in *triticum aestivum*. Int. J. Trop. Insect Sci..

[17] Javed K., Javed H., Mukhtar T., Qiu D. (2019). Pathogenicity of some entomopathogenic fungal strains to green peach aphid, *Myzus persicae* Sulzer (Homoptera: Aphididae). Egypt. J. Biol. Pest Control.

[18] Javed K., Javed H., Mukhtar T., Qiu D. (2019). Efficacy of *Beauveria bassiana* and *Verticillium lecanii* for the management of whitefly and aphid. Pak. J. Agric. Sci..

[19] Nouri-Ganbalani G., Borzoui E., Shahnavazi M., Nouri A. (2018). Induction of resistance against *Plutella xylostella* (L.) (Lepidoptera: Plutellidae) by jasmonic acid and mealy cabbage aphid feeding in *Brassica napus* L.. Front. Physiol..

[20] Salzman R.A., Brady J.A., Finlayson S.A., Buchanan C.D., Summer E.J., Sun F., Klein P.E., Klein R.R., Pratt L.H., Cordonnier-Pratt M.M. (2005). Transcriptional profiling of sorghum induced by methyl jasmonate, salicylic acid, and aminocyclopropane carboxylic acid reveals cooperative regulation and novel gene responses. Plant Physiol..

[21] Lazebnik J., Frago E., Dicke M., van Loon J.J.A. (2014). Phytohormone Mediation of Interactions Between Herbivores and Plant Pathogens. J. Chem. Ecol..

[22] Ali J.G., Agrawal A.A. (2014). Asymmetry of plant-mediated interactions between specialist aphids and caterpillars on two milkweeds. Funct. Ecol..

[23] Sandoya G.V., de Oliveira Buanafina M.M. (2014). Differential responses of Brachypodium distachyon genotypes to insect and fungal pathogens. Physiol. Mol. Plant Pathol..

[24] Wang H., Yang X., Guo L., Zeng H., Qiu D. (2015). PeBL1, a novel protein elicitor from *Brevibacillus laterosporus* strain A60, activates defense responses and systemic resistance in *Nicotiana benthamiana*. Appl. Environ. Microbiol..

[25] Jatoi G.H., Lihua G., Xiufen Y., Gadhi M.A., Keerio A.U., Abdulle Y.A., Qiu D. (2019). A novel protein elicitor PeBL2, from *Brevibacillus laterosporus* A60, induces systemic resistance against *Botrytis cinerea* in tobacco plant. Plant Pathol. J..

[26] Marche M.G., Mura M.E., Falchi G., Ruiu L. (2017). Spore surface proteins of *Brevibacillus laterosporus* are involved in insect pathogenesis. Sci. Rep..

[27] Barbieri G., Ferrari C., Mamberti S., Gabrieli P., Castelli M., Sassera D., Ursino E., Scoffone V.C., Radaelli G., Clementi E. (2021). Identification of a Novel *Brevibacillus laterosporus* Strain With Insecticidal Activity Against *Aedes albopictus* Larvae. Front. Microbiol..

[28] Bale J.S., Masters G.J., Hodkinson I.D., Awmack C., Bezemer T.M., Brown V.K., Butterfield J., Buse A., Coulson J.C., Farrar J. (2002). Herbivory in global climate change research: Direct effects of rising temperature on insect herbivores. Glob. Chang. Biol..

[29] Boughton A.J., Hoover K., Felton G.W. (2006). Impact of chemical elicitor applications on greenhouse tomato plants and population growth of the green peach aphid, *Myzus persicae*. Entomol. Exp. Appl..

[30] Mallinger R.E., Hogg D.B., Gratton C. (2011). Methyl Salicylate Attracts Natural Enemies and Reduces Populations of Soybean Aphids (Hemiptera: Aphididae) in Soybean Agroecosystems. J. Econ. Entomol..

[31] Schaller F., Schaller A., Stintzi A. (2004). Biosynthesis and metabolism of jasmonates. J. Plant Growth Regul..

[32] Glas J.J., Schimmel B.C.J., Alba J.M., Escobar-Bravo R., Schuurink R.C., Kant M.R. (2012). Plant glandular trichomes as targets for breeding or engineering of resistance to herbivores. Int. J. Mol. Sci..

[33] Tian D., Tooker J., Peiffer M., Chung S.H., Felton G.W. (2012). Role of trichomes in defense against herbivores: Comparison of herbivore response to woolly and hairless trichome mutants in tomato (*Solanum lycopersicum*). Planta.

[34] dos Santos Tozin L.R., Marques M.O.M., Rodrigues T.M. (2017). Herbivory by leaf-cutter ants changes the glandular trichomes density and the volatile components in an aromatic plant model. AoB Plants.

[35] Boughton A.J., Hoover K., Felton G.W. (2005). Methyl jasmonate application induces increased densities of glandular trichomes on tomato, *Lycopersicon esculentum*. J. Chem. Ecol..

[36] Ni Y., Wang J., Song C., Xia R.-E., Sun Z.-Y., Guo Y.-J., Li J.-N. (2013). Effects of SA Induction on Leaf Cuticular Wax and Resistance to Sclerotinia sclerotiorurn in *Brassica napus*. Acta Agron. Sin..

[37] Farmer E.E., Johnson R.R., Ryan C.A. (1992). Regulation of expression of proteinase inhibitor genes by methyl jasmonate and jasmonic acid. Plant Physiol..

[38] Mahmoud F., Mahfouz H. (2015). Effects of salicylic acid elicitor against aphids on wheat and detection of infestation using infrared thermal imaging technique in Ismailia, Egypt. Pestic. Phytomedicine/Pestic. I Fitomedicina.

[39] Cooper W.R., Goggin F.L. (2005). Effects of jasmonate-induced defenses in tomato on the potato aphid, *Macrosiphum euphorbiae*. Entomol. Exp. Appl..

[40] Hammond-Kosack K.E., Parker J.E. (2003). Deciphering plant-pathogen communication: Fresh perspectives for molecular resistance breeding. Curr. Opin. Biotechnol..

[41] Thaler J.S., Humphrey P.T., Whiteman N.K. (2012). Evolution of jasmonate and salicylate signal crosstalk. Trends Plant Sci..

[42] Kim H.S., Delaney T.P. (2002). Over-expression of TGA5, which encodes a bZIP transcription factor that interacts with NIM1/NPR1, confers SAR-independent resistance in *Arabidopsis thaliana* to *Peronospora parasitica*. Plant J..

[43] Chaerle L., Lenk S., Hagenbeek D., Buschmann C., Van Der Straeten D. (2007). Multicolor fluorescence imaging for early detection of the hypersensitive reaction to tobacco mosaic virus. J. Plant Physiol..

[44] Nicholson R.L., Hammerschmidt R. (1992). Phenolic Compounds And Their Role In Disease Resistance. Annu. Rev. Phytopathol..

[45] De Vos M., Van Oosten V.R., Van Poecke R.M.P., Van Pelt J.A., Pozo M.J., Mueller M.J., Buchala A.J., Métraux J.P., Van Loon L.C., Dicke M. (2005). Signal signature and transcriptome changes of *Arabidopsis* during pathogen and insect attack. Mol. Plant-Microbe Interact..

[46] Ali J.G., Agrawal A.A. (2012). Specialist versus generalist insect herbivores and plant defense. Trends Plant Sci..

[47] Li Y.H., Wei F., Dong X.Y., Peng J.H., Liu S.Y., Chen H. (2011). Simultaneous analysis of multiple endogenous plant hormones in leaf tissue of oilseed rape by solid-phase extraction coupled with high-performance liquid chromatography-electrospray ionisation tandem mass spectrometry. Phytochem. Anal..

[48] Jarošová J., Kundu J.K. (2010). Validation of reference genes as internal control for studying viral infections in cereals by quantitative real-time RT-PCR. BMC Plant Biol..

[49] Basit A., Farhan M., Essa M., Abbas M., Wang Y., Zhao D.G., Maridha A.U., Amjad Bashir M., Hussain A., Hanan A. (2021). Effect of two protein elicitors extracted from *Alternaria tenuissima* and *Beauveria bassiana* against rice leaf folder (*Marasmia exigua*). J. King Saud Univ. Sci..

[50] Livak K.J., Schmittgen T.D. (2001). Analysis of relative gene expression data using real-time quantitative PCR and the 2^-ΔΔCT^ method. Methods.

